# A commentary of “Consistency radio bursts in the Milky Way”: 10 remarkable discoveries from 2020 in *Nature*

**DOI:** 10.1016/j.fmre.2022.01.008

**Published:** 2022-02-01

**Authors:** Di Li

**Affiliations:** a National Astronomical Observatories, Chinese Academy of Sciences, Beijing, 100101, China; b NAOC-UKZN Computational Astrophysics Centre, University of KwaZulu-Natal, Durban, 4000, South Africa

## Abstract

The first detection and deep follow-up of a Galactic fast radio burst (FRB) phenomenon were reported in three papers published in the journal *Nature* in November 2020. Interestingly, this FRB is accompanied by a X-ray burst. The observations from multiple space and ground-based telescopes were combined to accomplish this discovery and ascertain its association with a source in the Milky Way. As the name implies, a FRB is a transient bright pulse of radio waves with a burst duration measured in milliseconds. This phenomenon was first discovered in 2007. It is extremely difficult to detect and even more so to determine their position in the sky due to their brief existence. This is the first detection of a FRB with radiation other than radio waves, as well as the first of its kind in the Milky Way. For the first time, these observations have confirmed that the source(s) of FRBs can be a magnetar(s), which so far is the only verified celestial body capable of producing FRBs. It is worth noting that one of the papers was written by a Chinese research team, the co-first authors of which are Lin Lin from Beijing Normal University, Chunfeng Zhang from Peking University, and Pei Wang from the National Astronomical Observatories of China. The observations came from China's “Sky Eye”—the Five-hundred-meter Aperture Spherical radio Telescope (FAST).①

The fast radio burst is a relatively new field. It was discovered in 2007, confirmed and named FRB in 2013, and localized to a certain host galaxy only in 2017, which for the first time confirmed FRB's cosmological origin. The American Astronomical Society dubbed this 2017 breakthrough “the most significant discovery in astronomy since the LIGO gravitational wave measurement.”

The origin of FRBs remains unknown. This unusual phenomenon lasts for about one-thousandth of a second and contains as much energy as that released by the whole Sun in days or even years. It has the potential to not only expand the horizon of fundamental physics and astrophysics but also provide a powerful tool for exploring the Universe. Lorimer et al. discovered the first FRB (FRB010724) when reprocessing the Parkes telescope's pulsar survey data of the Large and Small Magellanic Clouds. Hundreds of FRBs have since been detected, with more than a dozen confirmed host galaxies. The astronomical community has recognized the cosmological origin of FRB. A small number of FRB sources have shown repeated bursts. Will all FRBs recur? At the international FRB symposium held in March 2020, an informal vote held among the participating experts resulted in a nearly fifty-fifty split regarding the question. This reflects our current lack of understanding of this phenomenon.

On November 4, 2020, *Nature* published three papers on the first FRB in the Milky Way [1], [2], [3]. These three new observations help confirm that magnetars (neutron stars with extremely strong magnetic fields) are one of the sources of FRBs. The Canadian Hydrogen Intensity Surveying and Mapping Experiment (CHIME) telescope, the US STARE-2 telescope, and China's 500-m aperture spherical radio telescope “FAST” [4] contributed to these three papers. The first two papers establish the discovery, while the third provides in-depth monitoring. This result establishes the magnetar as the only celestial body capable of producing FRB-like bursts that have been observed and verified, laying the groundwork for unraveling various unusual phenomena surrounding FRB.

It is, therefore, a milestone.

Magnetars are a type of neutron star that has a lot of magnetism. The CHIME and STARE-2 telescopes were the first to detect the bright millisecond-level radio pulse, dubbed FRB 200428, toward the direction of the magnetar soft gamma-ray burst source SGR J1935+2154 in the Milky Way on April 28, 2020, and started to reveal the association between the FRB and the SGR source. Since late April, Chinese scientists have been monitoring it using international multi-band equipments, including FAST, the Fermi Satellite Gamma Burst Monitor (Fermi-GBM), optical BOOTES telescope, and China's Insight Hard X-ray Modulation Telescope (Insight-HXMT). No radio radiation was detected during the active period of source X and soft gamma-ray bursts, especially at the precise time nodes corresponding to the 29 soft gamma-ray bursts. The FAST measurements, combined with the detection of CHIME and STARE-2, covered a range of 8-order-of-magnitude in terms of brightness dynamic range, providing the most stringent radio limit of the FRB source in the Milky Way on the milli-Jansky flux level, thus providing important physical constraints of and important insights into the origin and physical mechanism of FRB. Through several narratives from various perspectives, Chinese and foreign research teams shared a story about the origins of FRB. Magnetars are the first celestial bodies to be confirmed to produce FRB-like bursts. The weak correlation between FRB and SGR bursts reflects the fact that the explosions of compact celestial bodies in the universe at different wavebands must rely on extremely rare physical conditions. This encourages more research into the radiation geometry and energy supply mechanism of magnetar FRBs, as well as pointing to a promising direction for understanding the physical origin of FRBs.

FRBs, given its extreme brightness, were discovered much later than what could be reasonably expected, with hindsight. Various radio surveys by large telescopes have been conducted for more than half a century, and this brightest transient source in the centimeter band of the universe could have even been detected by mass-produced TV antennas. The allure of the universe lies in its limitless possibilities. FAST has the best absolute sensitivity in history, which translates to great potential in detecting radio transient sources. FAST has discovered at least five new FRBs and detected thousands of bursts from repeating FRBs. It is making a unique contribution to understanding the mechanism of this mysterious cosmic phenomenon and advancing this new field of astronomy.

## Declaration of Competing Interest

The author declares that he does not have any conflicts of interest in this work.The author would like to disclose explicitly that he is a co-author of the papers discussed in this manuscript.
